# Supplementary material for: SMA-miRs (miR-181a-5p, -324-5p, and -451a) are overexpressed in spinal muscular atrophy skeletal muscle and serum samples
Source: eLife. 2021 Sep 20;10:e68054. doi: 10.7554/eLife.68054 (PMC8486378; doi:10.7554/eLife.68054)
Supplement: Supplementary file 1. [file elife-68054-supp1.docx]

**Supplementary Table 1. Demographic and genetic characteristics of subjects who underwent to muscle biopsy**

| **Sample** | **Sex** | **Age at biopsy (months)** | **SMN defect** |
| --- | --- | --- | --- |
| SMAI#1 | m | 7 | del/p.Tyr272Cys |
| SMAI#2 | f | 6 | del/del |
| SMAI#3 | m | 2 | del/del |
| SMAII#1 | m | 18 | del/del |
| SMAII#2 | m | 19 | del/del |
| SMAIII#1 | m | 217 | del/del |
| SMAIII#2 | f | 41 | del/del |
|  |  |  |  |
| CTRL#1 | m | 60 | - |
| CTRL#2 | m | 35 | - |
| CTRL#3 | m | 37 | - |
| CTRL#4 | m | 6 | - |
| CTRL#5 | m | 38 | - |
| CTRL#6 | m | 12 | - |
| CTRL#7 | m | 36 | - |
