# Supplementary material for: SMA-miRs (miR-181a-5p, -324-5p, and -451a) are overexpressed in spinal muscular atrophy skeletal muscle and serum samples
Source: eLife. 2021 Sep 20;10:e68054. doi: 10.7554/eLife.68054 (PMC8486378; doi:10.7554/eLife.68054)
Supplement: Supplementary file 2. [file elife-68054-supp2.docx]

**Supplementary Table 2: clinical and molecular characteristics of subjects included in the present study**

| **Patient #** | **Sex** | **Age^1^** | **SMA type** | | **SMN2 #** | ***SMN2*-fl** | **miR 324-5p** | **miR 181a-5p** | **miR 451a** | **miR sum** | **SMA-score** | **SMA-score** |
| --- | --- | --- | --- | --- | --- | --- | --- | --- | --- | --- | --- | --- |
|  |  | **(months)** |  | **(decimal classification)** |  | **(# of molecules/ng) ^2^** | **(# of molecules/µl) ^3^** | | | | **<72 months** | **>72 months** |
| 1 | f | 18.43 | 1 | 1.1 | 2 | 50.11 | 150.25 | 494.35 | 39.01 | 683.61 | 1.24 | 1.44 |
| 3 | m | 5.67 | 1 | 1.9 | 3 | 27.04 | 68.93 | 45.43 | 65.64 | 179.99 | 1.41 | 1.92 |
| 4 | m | 3.87 | 1 | 1.5 | 2 | 83.24 | 46.64 | 76.03 | 437.23 | 559.90 | 1.06 | 1.46 |
| 8 | f | 50.13 | 2 | 2.2 | 2 | 23.26 | 4625.56 | 153.58 | 187.03 | 4966.18 | 1.78 | 1.55 |
| 9 | f | 21.13 | 2 | 2 | 3 | 163.77 | 162.67 | 20.15 | 220.11 | 402.93 | 1.97 | 2.12 |
| 10 | m | 21.07 | 2 | 2.7 | 3 | 211.58 | 2459.69 | 305.47 | 218.20 | 2983.37 | 2.09 | 2.24 |
| 11 | m | 508.27 | 2 | NA | 2 | 26.02 | 543.98 | 53.78 | 24.43 | 622.18 |  | 2.05 |
| 12 | m | 32.37 | 2 | 2.1 | 3 | 113.15 | 153.51 | 66.01 | 60.64 | 280.15 | 2.06 | 2.07 |
| 13 | m | 57.43 | 2 | 2.7 | 3 | 120.60 | 519.56 | 42.01 | 26.77 | 588.34 | 2.52 | 2.12 |
| 14 | f | 439.80 | 2 | NA | 3 | 86.14 | 274.45 | 49.55 | 29.17 | 353.17 |  | 2.57 |
| 15 | f | 21.93 | 2 | 2.3 | 3 | 192.70 | 164.23 | 350.72 | 544.25 | 1059.19 | 2.05 | 2.17 |
| 16 | m | 21.93 | 2 | 2.6 | 3 | 99.78 | 272.17 | 122.19 | 32.84 | 427.19 | 1.85 | 2.04 |
| 17 | f | 32.93 | 2 | 2.5 | 3 | 109.01 | 170.76 | 102.15 | 51.80 | 324.71 | 2.06 | 2.06 |
| 2 | f | 84.33 | 2 | 2.2 | 3 | 95.69 | 177.35 | 176.11 | 29.22 | 382.69 |  | 2.12 |
| 18 | f | 14.43 | 2 | 2.1 | 3 | 281.72 | 132.99 | 670.08 | 242.68 | 1045.75 | 2.11 | 2.28 |
| 19 | f | 21.37 | 2 | 2.2 | 3 | 206.88 | 129.50 | 144.01 | 29.85 | 303.37 | 2.07 | 2.17 |
| 20 | f | 38.43 | 2 | 2.3 | 3 | 49.84 | 6797.29 | 441.14 | 129.84 | 7368.27 | 2.09 | 2.16 |
| 21 | f | 17.07 | 2 | 2 | 2 | 216.49 | 286.83 | 72.87 | 44.22 | 403.92 | 1.57 | 1.64 |
| 22 | m | 25.47 | 2 | 2.3 | 3 | 340.77 | 1631.90 | 279.98 | 207.16 | 2119.04 | 2.44 | 2.39 |
| 23 | m | 31.07 | 2 | 2.1 | 3 | 98.31 | 66.97 | 128.44 | 43.21 | 238.61 | 2.00 | 2.04 |
| 24 | m | 16.20 | 2 | 2 | 3 | 31.33 | 198.60 | 372.71 | 33.11 | 604.41 | 1.60 | 1.95 |
| 25 | f | 17.37 | 2 | 2.3 | 3 | 196.21 | 228.05 | 573.51 | 567.14 | 1368.70 | 1.98 | 2.18 |
| 26 | m | 23.20 | 2 | 3 | 3 | NA | 445.52 | 125.56 | 29.33 | 600.41 |  |  |
| 30 | m | 28.27 | 2 | NA | 3 | NA | NA | 17.47 | 25.46 | NA |  |  |
| 31 | m | 17.33 | 2 | NA | 3 | NA | NA | 10.58 | 4.25 | NA |  |  |
| 32 | f | 56.57 | 3 | 3.5 | 4 | 160.13 | 1198.04 | 934.36 | 347.38 | 2479.78 | 3.04 | 2.75 |
| 33 | m | 203.77 | 3 | 3.5 | 4 | 88.11 | 672.48 | 50.81 | 17.24 | 740.54 |  | 2.81 |
| 34 | f | 76.07 | 3 | 3.5 | 3 | 30.40 | 160.23 | 503.39 | 30.63 | 694.25 |  | 2.03 |
| 35 | m | 225.37 | 3 | 3.5 | 4 | 56.35 | 2593.56 | 113.82 | 33.27 | 2740.66 |  | 2.85 |
| 36 | m | 372.20 | 3 | 3 | 3 | 150.90 | 651.49 | 59.49 | 67.39 | 778.37 |  | 2.57 |
| 37 | f | 234.63 | 3 | 3.5 | 4 | 142.92 | 114.30 | 119.88 | 12.76 | 246.94 |  | 2.91 |
| 38 | m | 678.70 | 3 | 3.5 | NA | 58.99 | 1854.07 | 229.25 | 109.10 | 2192.42 |  |  |
| 39 | f | 31.07 | 3 | 3 | 4 | 178.38 | 98.67 | 256.04 | 71.98 | 426.69 | 2.62 | 2.69 |
| 40 | m | 445.97 | 3 | 3.5 | 4 | 85.68 | 150.13 | 74.26 | 368.87 | 593.27 |  | 3.13 |
| 41 | f | 637.03 | 3 | 3.5 | 4 | 94.07 | 2400.93 | 408.14 | 227.54 | 3036.62 |  | 3.45 |
| 42 | m | 636.60 | 3 | 3.5 | 4 | 63.37 | 99.06 | 85.22 | 415.32 | 599.60 |  | 3.35 |
| 43 | f | 724.27 | 3 | NA | NA | 44.15 | 920.74 | 322.44 | 425.90 | 1669.08 |  |  |
| 44 | m | 333.70 | 3 | 3 | NA | 323.67 | 559.34 | 47.86 | 41.98 | 649.18 |  |  |
| 45 | f | 37.20 | 3 | 3 | 3 | 221.24 | 173.78 | 697.89 | 828.73 | 1700.40 | 2.39 | 2.25 |
| 46 | m | 473.60 | 3 | 3.5 | 4 | 96.73 | 4040.02 | 481.03 | 299.98 | 4821.03 |  | 3.28 |
| 47 | f | 306.37 | 3 | 3.5 | 4 | 160.94 | 200.53 | 38.81 | 36.92 | 276.25 |  | 3.03 |
| 48 | f | 342.43 | 3 | 3.5 | 4 | 223.00 | 378.81 | 227.28 | 47.41 | 653.50 |  | 3.17 |
| 49 | f | 357.03 | 3 | 3 | 3 | 72.15 | 211.87 | 651.71 | 271.90 | 1135.48 |  | 2.46 |
| 50 | f | 443.77 | 3 | 3.5 | 4 | 70.86 | 30.53 | 77.44 | 109.11 | 217.08 |  | 3.09 |
| 51 | f | 367.90 | 3 | 3.5 | 3 | 192.07 | 415.83 | 85.57 | 4.81 | 506.22 |  | 2.61 |
| 52 | m | 430.03 | 3 | 3.5 | NA | 118.39 | 77.82 | 126.00 | 269.01 | 472.83 |  |  |
| 53 | f | 159.30 | 3 | 3 | 3 | 99.54 | 157.15 | 265.08 | 286.18 | 708.41 |  | 2.23 |
| 54 | m | 52.73 | 3 | NA | 3 | NA | NA | 19.47 | 391.59 | NA |  |  |
| 55 | f | 43.63 | 3 | NA | 3 | NA | NA | 14.14 | 72.13 | NA |  |  |
| 57 | f | 252.77 | 3 | NA | 3 | NA | NA | 2.72 | 299.62 | NA |  |  |
| 58 | f | 755.53 | 4 | 4 | 4 | 143.94 | 164.46 | 537.73 | 382.44 | 1084.63 |  | 3.62 |
|  |  |  |  |  |  |  |  |  |  |  |  |  |
| **Control #** |  |  |  |  |  |  |  |  |  |  |  |  |
| 1 | f | 311.67 |  |  |  |  | 177.77 | 46.59 | 18.92 | 243.28 |  |  |
| 2 | f | 167.67 |  |  |  |  | 213.25 | 75.29 | 0.00 | 288.54 |  |  |
| 3 | f | 97.33 |  |  |  |  | 1858.97 | 67.99 | 4.61 | 1931.57 |  |  |
| 4 | m | 94.60 |  |  |  |  | 477.29 | 62.18 | 14.67 | 554.15 |  |  |
| 5 | f | 56.00 |  |  |  |  | 217.21 | 46.86 | 21.94 | 286.01 |  |  |
| 6 | m | 66.67 |  |  |  |  | 126.22 | 102.12 | 26.73 | 255.07 |  |  |
| 7 | m | 58.90 |  |  |  |  | 100.02 | 170.57 | 0.00 | 270.59 |  |  |
| 8 | m | 46.47 |  |  |  |  | 144.24 | 0.00 | 0.00 | 144.24 |  |  |
| 9 | f | 48.73 |  |  |  |  | 119.91 | 293.64 | 0.00 | 413.55 |  |  |
| 10 | m | 47.73 |  |  |  |  | 268.70 | 79.97 | 5.50 | 354.17 |  |  |
| 11 | m | 48.60 |  |  |  |  | 613.37 | 52.83 | 0.00 | 666.20 |  |  |
| 12 | f | 39.93 |  |  |  |  | 181.94 | 104.07 | 91.25 | 377.26 |  |  |
| 13 | f | 32.33 |  |  |  |  | 547.49 | 95.10 | 306.32 | 948.90 |  |  |
| 14 | f | 22.33 |  |  |  |  | 0.00 | 325.24 | 0.00 | 325.24 |  |  |
| 15 | f | 27.23 |  |  |  |  | 237.26 | 53.07 | 16.68 | 307.02 |  |  |
| 16 | m | 21.70 |  |  |  |  | 298.60 | 173.61 | 576.05 | 1048.26 |  |  |
| 17 | m | 160.50 |  |  |  |  | 136.77 | 87.72 | 160.93 | 385.42 |  |  |
| 18 | f | 119.20 |  |  |  |  | 186.07 | 93.81 | 16.62 | 296.50 |  |  |
| 19 | m | 108.27 |  |  |  |  | 131.73 | 50.28 | 18.88 | 200.89 |  |  |
| 20 | m | 108.70 |  |  |  |  | 545.98 | 43.43 | 0.00 | 589.41 |  |  |
| 21 | f | 327.40 |  |  |  |  | 160.46 | 41.21 | 0.00 | 201.67 |  |  |
| 22 | f | 362.27 |  |  |  |  | 90.75 | 48.53 | 18.92 | 158.20 |  |  |
| 23 | f | 341.93 |  |  |  |  | 40.24 | 96.12 | 30.59 | 166.95 |  |  |
| 24 | m | 316.57 |  |  |  |  | 122.58 | 33.80 | 0.51 | 156.88 |  |  |
| 25 | m | 168.43 |  |  |  |  | 196.28 | 422.30 | 261.82 | 880.40 |  |  |
| 26 | m | 67.33 |  |  |  |  | 116.51 | 18.71 | 166.90 | 302.13 |  |  |
| 27 | f | 61.50 |  |  |  |  | 43.47 | 60.77 | 6.97 | 111.21 |  |  |
| 28 | m | 36.97 |  |  |  |  | 24.27 | 5.42 | 40.03 | 69.71 |  |  |
| 29 | f | 15.53 |  |  |  |  | 50.91 | 21.24 | 38.83 | 110.98 |  |  |
| 30 | f | 34.33 |  |  |  |  | 18.95 | 0.00 | 25.79 | 44.74 |  |  |
| 31 | m | 173.13 |  |  |  |  | 24.97 | 0.00 | 10.52 | 35.49 |  |  |
| 32 | m | 96.53 |  |  |  |  | 0.00 | 5.79 | 48.77 | 54.56 |  |  |
| 33 | m | 84.77 |  |  |  |  | 0.00 | 8.10 | 74.48 | 82.59 |  |  |
| 34 | f | 315.67 |  |  |  |  | 0.00 | 33.94 | 309.39 | 343.32 |  |  |
| 35 | f | 387.07 |  |  |  |  | 0.00 | 22.70 | 155.04 | 177.74 |  |  |
| 36 | f | 330.17 |  |  |  |  | 0.00 | 12.28 | 7.69 | 19.97 |  |  |
| 37 | m | 304.83 |  |  |  |  | 0.00 | 1.98 | 130.65 | 132.63 |  |  |

^1^: Age at sampling

^2^: # of molecules/ng of total RNA

^3^: # of molecules/µl of serum

NA: not available
