# Supplementary material for: SMA-miRs (miR-181a-5p, -324-5p, and -451a) are overexpressed in spinal muscular atrophy skeletal muscle and serum samples
Source: eLife. 2021 Sep 20;10:e68054. doi: 10.7554/eLife.68054 (PMC8486378; doi:10.7554/eLife.68054)
Supplement: Supplementary file 3. [file elife-68054-supp3.docx]

**Supplementary Table 3: Primer sequences**

| **Primer name** | **Sequence** |
| --- | --- |
| hsa-miR-let-7c-F | 5’-CGACTGACTTGAGGTAGTAGGTTGTATGGTT-3’ |
| hsa-miR-1-F | 5'-GACTGACTGACTTGGAATGTAAAGAAGTATGTAT-3' |
| hsa-miR-122-5p-F | 5'-CTGACTTGGAGTGTGACAATGGTGTTTG-3' |
| hsa-miR-1273g-3p-F | 5’-ACCACTGCACTCCAGCCTGAG-3’ |
| hsa-miR-1281-F | 5’-CTTCGCCTCCTCCTCTCCC-3’ |
| hsa-miR-1303-F | 5’-GACTTTTAGAGACGGGGTCTTGCTCT-3’ |
| hsa-miR-133b-F | 5’-GACTTTTGGTCCCCTTCAACCAGCTA-3’ |
| hsa-miR-143-3p-F | 5’-GACTGACTTGAGATGAAGCACTGTAGCTC-3’ |
| hsa-miR-145-5p-F | 5’-CTGTCCAGTTTTCCCAGGAATCCCT-3’ |
| hsa-miR-1469-F | 5’-CTCGGCGCGGGGC-3’ |
| hsa-miR-146a-5p-F | 5’-GACTGACTTGAGAACTGAATTCCATGGGTT-3’ |
| hsa-miR-146b-5p-F | 5’-GACTGACTTGAGAACTGAATTCCATAGGCT-3’ |
| hsa-miR-150-5p-F | 5'-GACTTCTCCCAACCCTTGTACCAGTG-3' |
| hsa-miR-16a-5p-F | 5'-ACTGACTTAGCAGCACGTAAATATTGGCG-3' |
| hsa-miR-181a-5p-F | 5’-TCAACATTCAACGCTGTCGGTGAGT-3’ |
| hsa-miR-19a-3p-F | 5’-GACTGACTTGTGCAAATCTATGCAAAACTGA-3’ |
| hsa-miR-19b-3p-F | 5’-CTGACTTGTGCAAATCCATGCAAAACTGA-3’ |
| hsa-miR-206-F | 5’-GACTGACTTGGAATGTAAGGAAGTGTGTGG-3’ |
| hsa-miR-215-5p-F | 5’-GACTGACTGACTATGACCTATGAATTGACAGAC-3’ |
| hsa-miR-23a-3p-F | 5’-GACTGACTATCACATTGCCAGGGATTTCC-3’ |
| hsa-miR-26a-5p-F | 5'-GACTGACTTTCAAHTAATCCAGGATAGGCT-3' |
| hsa-miR-29b-1-5p-F | 5'-CTGACTGCTGGTTTCATATGGTGGTTTAGA-3' |
| hsa-miR-30c-5p-F | 5'-GACTGACTTGTAAACATCCTACACTCTCAGC-3' |
| hsa-miR-3196-F | 5’-CGGGGCGGCAGGG-3’ |
| hsa-miR-324-5p-F | 5’-CGCATCCCCTAGGGCATTGGTGT-3’ |
| hsa-miR-329-3p-F | 5'-TGACTGACTAACACACCTGGTTAACCTCTTT-3' |
| hsa-miR-329-5p-F | 5'-ACTGACTGAGGTTTTCTGGGTTTCTGTTTC-3' |
| hsa-miR-335-5p-F | 5’-ACTGACTGACTTCAAGAGCAATAACGAAAAATGT-3’ |
| hsa-miR-339-5p-F | 5'-TCCCTGTCCTCCAGGAGCTCACG-3' |
| hsa-miR-3591-5p-F | 5’-CTGACTGACTTTTAGTGTGATAATGGCGTTTGA-3’ |
| hsa-miR-3605-3p-F | 5’-CTCCTCCGTGTTACCTGTCCTCTAG-3’ |
| hsa-miR-3613-3p-F | 5’-GACTGACTACAAAAAAAAAAGCCCAACCCTTC-3’ |
| hsa-miR-362-3p-F | 5’-ACTGACTGACTAACACACCTATTCAAGGATTCA-3’ |
| hsa-miR-376c-3p-F | 5’-TCGACTGACTAACATAGAGGAAATTCCACGT-3’ |
| hsa-miR-378d-F | 5’-GACTGACTACTGGACTTGGAGTCAGAAA-3’ |
| hsa-miR-378e-F | 5’-CTGACTACTGGACTTGGAGTCAGGA-3’ |
| hsa-miR-378g-F | 5’-CTGACTACTGGGCTTGGAGTCAGAAG-3’ |
| hsa-miR-3909-F | 5’-TGTCCTCTAGGGCCTGCAGTC-3’ |
| hsa-miR-3913-5p-F | 5’-GACTGACTTTTGGGACTGATCTTGATGTCT-3’ |
| hsa-miR-3960-F | 5’-GGCGGCGGCGGA-3’ |
| hsa-miR-4443-F | 5’-CTGACTTTGGAGGCGTGGGTTTT-3’ |
| hsa-miR-4454-F | 5’-GGATCCGAGTCACGGCACC-3’ |
| hsa-miR-451a-F | 5’-ACTGACTGACTAAACCGTTACCATTACTGAGTT-3’ |
| hsa-miR-4532-F | 5’-CCCCGGGGAGCCCGGCG-3’ |
| hsa-miR-4770-F | 5’-CTGACTGACTTGAGATGACACTGTAGCT-3’ |
| hsa-miR-4800-3p-F | 5’-CTCATCCGTCCGTCTGTCCAC-3’ |
| hsa-miR-499a-3p-F | 5’-CTGACTAACATCACAGCAAGTCTGTGCT-3’ |
| hsa-miR-542-3p-F | 5’-GACTGACTGACTTGTGACAGATTGATAACTGAAA-3’ |
| hsa-miR-5690-F | 5’-GACTGACTGACTTCAGCTACTACCTCTATTAGG-3’ |
| hsa-miR-6500-3p-F | 5’-GACTACACTTGTTGGGATGACCTGC-3’ |
| hsa-miR-6724-5p-F | 5’-CTGGGCCCGCGGC-3’ |
| hsa-miR-9-3p-F | 5'-CTGACTGACTGACTGAATAAAGCTAGATAACCGAAAGT-3' |
| Universal-Reverse-Primer | 5’-CTGACTGACTGACACGGAGGTACTAG-3’ |
| hsa-miR-181a-5p-gDNA-F | 5'-ACAGTTCAACCCACCGACA-3' |
| hsa-miR-181a-5p-gDNA-R | 5'-CGGCCATGTTTTTGCTTAAT-3' |
| hsa-miR-324-5p-gDNA-F | 5'-AGCTGGCACGGATGGTTAT-3' |
| hsa-miR-324-5pa-gDNA-R | 5'-TTGTTTTCTTAAAAGGGGTGGA-3' |
| hsa-miR-451a-gDNA-F | 5'-GTATCTATTCCCTCCCCTACCC-3' |
| hsa-miR-451a-gDNA-R | 5'-CTCTGGAGCCTGACAAGGAG-3' |
| hsa-let7cES-F | 5'-TGAGGTAGTAGGTTGTATGGTTAAAAAAAAAAAAAAACTA-3' |
| hsa-let7cES-R | 5'-TGACACGGAGGTACTAGTTTTTTTTTTTTTTTAACC-3' |
| hsa-miR-1ES-F | 5'-TGGAATGTAAAGAAGTATGTATAAAAAAAAAAAAAAACTA-3' |
| hsa-miR-1ES-R | 5'-TGACACGGAGGTACTAGTTTTTTTTTTTTTTTATAT-3' |
| hsa-miR-122-5pES-F | 5'-TGGAGTGTGACAATGGTGTTTGAAAAAAAAAAAAAAACTA-3' |
| hsa-miR-122-5pES-R | 5'-TGACACGGAGGTACTAGTTTTTTTTTTTTTTTCAAA-3' |
| hsa-miR-1273g-3pES-F | 5'-ACCACTGCACTCCAGCCTGAGAAAAAAAAAAAAAAACTA-3' |
| hsa-miR-1281ES-F | 5'-TCGCCTCCTCCTCTCCCAAAAAAAAAAAAAAACTA-3' |
| hsa-miR-1281ES-R | 5'-TGACACGGAGGTACTAGTTTTTTTTTTTTTTTGG-3' |
| hsa-miR-1303ES-F | 5'-TTTAGAGACGGGGTCTTGCTCTAAAAAAAAAAAAAAACTA-3' |
| hsa-miR-1303ES-R | 5'-TGACACGGAGGTACTAGTTTTTTTTTTTTTTTAG-3' |
| hsa-miR-133bES-F | 5'-TTTGGTCCCCTTCAACCAGCTAAAAAAAAAAAAAAAACTA-3' |
| hsa-miR-133bES-R | 5'-TGACACGGAGGTACTAGTTTTTTTTTTTTTTTAGCT-3' |
| hsa-miR-143-3pES-F | 5'-GGTGCAGTGCTGCATCTCTGGTAAAAAAAAAAAAAAACTA-3' |
| hsa-miR-143-3pES-R | 5'-TGACACGGAGGTACTAGTTTTTTTTTTTTTTTACCA-3' |
| hsa-miR-145-5pES-F | 5'-GTCCAGTTTTCCCAGGAATCCCTAAAAAAAAAAAAAAACTA -3' |
| hsa-miR-145-5pES-R | 5'-TGACACGGAGGTACTAGTTTTTTTTTTTTTTTAGGG-3' |
| hsa-miR-1469ES-F | 5'-CTCGGCGCGGGGCGCGGGCTCCAAAAAAAAAAAAAAACTA-3' |
| hsa-miR-146a-5pES-F | 5'-TGAGAACTGAATTCCATGGGTTAAAAAAAAAAAAAAACTA -3' |
| hsa-miR-146a-5pES-R | 5'-TGACACGGAGGTACTAGTTTTTTTTTTTTTTTAACC-3' |
| hsa-miR-146b-5pES-F | 5'-TGAGAACTGAATTCCATAGGCTAAAAAAAAAAAAAAACTA-3' |
| hsa-miR-146b-5pES-R | 5'-TGACACGGAGGTACTAGTTTTTTTTTTTTTTTAGCC-3' |
| hsa-miR-15b-3pES-F | 5'-CGAATCATTATTTGCTGCTCTAAAAAAAAAAAAAAAACTA-3' |
| hsa-miR-15b-3pES-R | 5'-TGACACGGAGGTACTAGTTTTTTTTTTTTTTTTAGA-3' |
| hsa-miR-150-5pES-F | 5'-TCTCCCAACCCTTGTACCAGTGAAAAAAAAAAAAAAACTA-3' |
| hsa-miR-150-5pES-R | 5'-TGACACGGAGGTACTAGTTTTTTTTTTTTTTTCACT-3' |
| hsa-miR-16a-5pES-F | 5'-TTAGCAGCACGTAAATATTGGCGAAAAAAAAAAAAAAACTA-3 |
| hsa-miR-16a-5pES-R | 5'-TGACACGGAGGTACTAGTTTTTTTTTTTTTTTCGCC-3' |
| hsa-miR-181a-5pES-F | 5'-AACATTCAACGCTGTCGGTGAGTAAAAAAAAAAAAAAACTA-3' |
| hsa-miR-181a-5pES-R | 5'- TGACACGGAGGTACTAGTTTTTTTTTTTTTTTACT-3' |
| hsa-miR-19a-3pES-F | 5'-TGTGCAAATCTATGCAAAACTGAAAAAAAAAAAAAAAACTA-3' |
| hsa-miR-19b-3pES-F | 5'-TGTGCAAATCCATGCAAAACTGAAAAAAAAAAAAAAAACTA-3' |
| hsa-miR-19b-3pES-R | 5'-TGACACGGAGGTACTAGTTTTTTTTTTTTTTTTCAG -3' |
| hsa-miR-206ES-F | 5'-TGGAATGTAAGGAAGTGTGTGGAAAAAAAAAAAAAAACTA-3' |
| hsa-miR-206ES-R | 5'-TGACACGGAGGTACTAGTTTTTTTTTTTTTTTCCAC-3' |
| hsa-miR-215-5pES-F | 5'-ATGACCTATGAATTGACAGACAAAAAAAAAAAAAAACTA-3' |
| hsa-miR-215-5pES-R | 5'-TGACACGGAGGTACTAGTTTTTTTTTTTTTTTGTCT-3' |
| hsa-miR-215-3pES-F | 5'-TCTGTCATTTCTTTAGGCCAATAAAAAAAAAAAAAAAACTA-3' |
| hsa-miR-215-3pES-R | 5'-TGACACGGAGGTACTAGTTTTTTTTTTTTTTTTATT-3' |
| hsa-miR-23a-3pES-F | 5'-ATCACATTGCCAGGGATTTCCAAAAAAAAAAAAAAACTA-3' |
| hsa-miR-23a-3pES-R | 5'-TGACACGGAGGTACTAGTTTTTTTTTTTTTTTGGAA-3' |
| hsa-miR-26a-5pES-F | 5'-TTCAAGTAATCCAGGATAGGCTAAAAAAAAAAAAAAACTA-3' |
| hsa-miR-26a-5pES-R | 5'-TGACACGGAGGTACTAGTTTTTTTTTTTTTTTAACC-3' |
| hsa-miR-29b-1-5pES-F | 5'-GCTGGTTTCATATGGTGGTTTAGAAAAAAAAAAAAAAAACTA-3' |
| hsa-miR-29b-1-5pES-R | 5'-TGACACGGAGGTACTAGTTTTTTTTTTTTTTTTCTA-3' |
| hsa-miR-30c-5pES-F | 5'-TGTAAACATCCTACACTCTCAGCAAAAAAAAAAAAAAAACTA-3' |
| hsa-miR-30c-5pES-R | 5'-TGACACGGAGGTACTAGTTTTTTTTTTTTTTTGCTG-3' |
| hsa-miR-3196ES-F | 5'-CGGGGCGGCAGGGGCCTCAAAAAAAAAAAAAAACTA-3' |
| hsa-miR-3196ES-R | 5'-TGACACGGAGGTACTAGTTTTTTTTTTTTTTTGA-3' |
| hsa-miR-324-5pES-F | 5'-CGCATCCCCTAGGGCATTGGTGAAAAAAAAAAAAAAACTA-3' |
| hsa-miR-324-5pES-R | 5'-TGACACGGAGGTACTAGTTTTTTTTTTTTTTTCACC-3' |
| hsa-miR-329-3pES-F | 5'-AACACACCTGGTTAACCTCTTTAAAAAAAAAAAAAAACTA-3' |
| hsa-miR-329-3pES-R | 5'-TGACACGGAGGTACTAGTTTTTTTTTTTTTTTAAAG-3' |
| hsa-miR-329-5pES-F | 5'-GAGGTTTTCTGGGTTTCTGTTTCAAAAAAAAAAAAAAACTA-3' |
| hsa-miR-329-5pES-R | 5'-TGACACGGAGGTACTAGTTTTTTTTTTTTTTTGAAA-3' |
| hsa-miR-335-5pES-F | 5'-TCAAGAGCAATAACGAAAAATGTAAAAAAAAAAAAAAACTA-3' |
| hsa-miR-335-5pES-R | 5'-TGACACGGAGGTACTAGTTTTTTTTTTTTTTTACAT-3' |
| hsa-miR-339-5pES-F | 5'-TCCCTGTCCTCCAGGAGCTCACGAAAAAAAAAAAAAAACTA-3' |
| hsa-miR-339-5pES-R | 5'-TGACACGGAGGTACTAGTTTTTTTTTTTTTTTCGTG-3' |
| hsa-miR-3591-5pES-F | 5'-TTTAGTGTGATAATGGCGTTTGAAAAAAAAAAAAAAAACTA-3' |
| hsa-miR-3605-3pES-F | 5'-CCTCCGTGTTACCTGTCCTCTAGAAAAAAAAAAAAAAACTA-3' |
| hsa-miR-3613-3pES-F | 5'-ACAAAAAAAAAAGCCCAACCCTTCAAAAAAAAAAAAAAACTA-3' |
| hsa-miR-362-3pES-F | 5'-AACACACCTATTCAAGGATTCAAAAAAAAAAAAAAAACTA-3' |
| hsa-miR-362-3pES-R | 5'-TGACACGGAGGTACTAGTTTTTTTTTTTTTTTTGAA-3' |
| hsa-miR-376c-3pES-F | 5'-AACATAGAGGAAATTCCACGTAAAAAAAAAAAAAAACTA-3' |
| hsa-miR-376c-3pES-R | 5'-TGACACGGAGGTACTAGTTTTTTTTTTTTTTTACGT-3' |
| hsa-miR-378dES-F | 5'-ACTGGACTTGGAGTCAGAAAAAAAAAAAAAAAAAACTA-3' |
| hsa-miR-378dES-R | 5'-TGACACGGAGGTACTAGTTTTTTTTTTTTTTTTTTC-3' |
| hsa-miR-378eES-F | 5'-ACTGGACTTGGAGTCAGGAAAAAAAAAAAAAAAACTA-3' |
| hsa-miR-378eES-R | 5'-TGACACGGAGGTACTAGTTTTTTTTTTTTTTTTC-3' |
| hsa-miR-378gES-F | 5'-ACTGGGCTTGGAGTCAGAAGAAAAAAAAAAAAAAACTA-3' |
| hsa-miR-378gES-R | 5'-TGACACGGAGGTACTAGTTTTTTTTTTTTTTTCT-3' |
| hsa-miR-3909ES-F | 5'-TGTCCTCTAGGGCCTGCAGTCTAAAAAAAAAAAAAAACTA-3' |
| hsa-miR-3913-5pES-F | 5'-TTTGGGACTGATCTTGATGTCTAAAAAAAAAAAAAAACTA-3' |
| hsa-miR-3960ES-F | 5'-GGCGGCGGCGGAGGCGGGGGAAAAAAAAAAAAAAACTA-3' |
| hsa-miR-3960ES-R | 5'-TGACACGGAGGTACTAGTTTTTTTTTTTTTTTCC -3' |
| hsa-miR-4443ES-F | 5'-TTGGAGGCGTGGGTTTTAAAAAAAAAAAAAAACTA-3' |
| hsa-miR-4443ES-R | 5'-TGACACGGAGGTACTAGTTTTTTTTTTTTTTTAAAA-3' |
| hsa-miR-4454ES-F | 5'-GGATCCGAGTCACGGCACCAAAAAAAAAAAAAAAACTA-3' |
| hsa-miR-4454ES-R | 5'-TGACACGGAGGTACTAGTTTTTTTTTTTTTTTTGG-3' |
| hsa-miR-451aES-F | 5'-AAACCGTTACCATTACTGAGTTAAAAAAAAAAAAAAACTA-3' |
| hsa-miR-451aES-R | 5'-TGACACGGAGGTACTAGTTTTTTTTTTTTTTTAACT-3' |
| hsa-miR-4532ES-F | 5'-CCCCGGGGAGCCCGGCGAAAAAAAAAAAAAAACTA-3' |
| hsa-miR-4532ES-R | 5'-TGACACGGAGGTACTAGTTTTTTTTTTTTTTTCGCC-3' |
| hsa-miR-4770ES-F | 5'-TGAGATGACACTGTAGCTAAAAAAAAAAAAAAACTA-3' |
| hsa-miR-4800-3pES-F | 5'-CATCCGTCCGTCTGTCCACAAAAAAAAAAAAAAACTA-3' |
| hsa-miR-4800-3pES-R | 5'-TGACACGGAGGTACTAGTTTTTTTTTTTTTTTGTGG-3' |
| hsa-miR-499a-3pES-F | 5'-AACATCACAGCAAGTCTGTGCTAAAAAAAAAAAAAAACTA-3' |
| hsa-miR-499a-3pES-R | 5'-TGACACGGAGGTACTAGTTTTTTTTTTTTTTTAGCA-3' |
| hsa-miR-542-3pES-F | 5'-TGTGACAGATTGATAACTGAAAAAAAAAAAAAAAAAACTA-3' |
| hsa-miR-5690ES-F | 5'-TCAGCTACTACCTCTATTAGGAAAAAAAAAAAAAAACTA-3' |
| hsa-miR-6500-3pES-F | 5'-ACACTTGTTGGGATGACCTGCAAAAAAAAAAAAAAACTA-3' |
| hsa-miR-6500-3pES-R | 5'-TGACACGGAGGTACTAGTTTTTTTTTTTTTTTGCAG-3' |
| hsa-miR-6724-5pES-F | 5'-CTGGGCCCGCGGCGGGCGTGGGGAAAAAAAAAAAAAAACTA-3' |
| hsa-miR-9-3pES-F | 5'-ATAAAGCTAGATAACCGAAAGTAAAAAAAAAAAAAAAAACTA-3' |
| hsa-miR-9-3pES-R | 5'-TGACACGGAGGTACTAGTTTTTTTTTTTTTTTACTT-3' |
