# Supplementary material for: SMA-miRs (miR-181a-5p, -324-5p, and -451a) are overexpressed in spinal muscular atrophy skeletal muscle and serum samples
Source: eLife. 2021 Sep 20;10:e68054. doi: 10.7554/eLife.68054 (PMC8486378; doi:10.7554/eLife.68054)
Supplement: Supplementary file 4. [file elife-68054-supp4.docx]

**Supplementary Table 4: List of deregulated miRs in whole miRNome analyses (patients vs. controls)**

| **miRNAs differentially expressed in muscle biopsies** | | | | |
| --- | --- | --- | --- | --- |
| **hsa-miR** | **LogFC** | **P-value** | **FDR** | **logCPM** |
| miR-205-5p | 3.70 | 1.81E-11 | 9.44E-09 | 1.85 |
| miR-499a-3p§ | -3.07 | 5.21E-09 | 1.36E-06 | 3.01 |
| miR-19a-3p | 2.65 | 1.17E-08 | 2.04E-06 | 6.73 |
| miR-184 | 3.30 | 5.30E-08 | 5.04E-06 | 1.03 |
| miR-4532 | -2.47 | 5.35E-08 | 5.04E-06 | 8.29 |
| miR-215 | 2.56 | 5.82E-08 | 5.04E-06 | 3.36 |
| miR-95 | -2.66 | 1.20E-07 | 8.93E-06 | 6.89 |
| miR-224-5p | 2.49 | 1.65E-07 | 1.07E-05 | 4.83 |
| miR-19b-3p | 2.34 | 3.57E-07 | 1.89E-05 | 9.11 |
| miR-655 | 2.90 | 3.64E-07 | 1.89E-05 | 2.15 |
| miR-452-5p | 2.41 | 4.45E-07 | 2.10E-05 | 4.48 |
| miR-338-5p | 2.19 | 7.66E-07 | 3.32E-05 | 4.23 |
| miR-5690 | -2.89 | 1.07E-06 | 4.28E-05 | 1.06 |
| miR-1469 | -2.54 | 1.20E-06 | 4.47E-05 | 2.20 |
| miR-885-5p | -2.36 | 3.93E-06 | 1.36E-04 | 4.11 |
| miR-335-5p | 2.08 | 7.78E-06 | 2.53E-04 | 7.71 |
| miR-204-5p | 1.96 | 8.71E-06 | 2.67E-04 | 6.51 |
| miR-145-5p | 1.76 | 1.65E-05 | 4.78E-04 | 10.13 |
| miR-1303 | -2.38 | 2.27E-05 | 6.20E-04 | 1.44 |
| miR-299-5p | 2.38 | 2.64E-05 | 6.87E-04 | 3.44 |
| miR-512-3p | -2.11 | 2.98E-05 | 7.37E-04 | 1.91 |
| miR-218-5p | 1.91 | 3.24E-05 | 7.37E-04 | 4.21 |
| miR-1281 | -1.95 | 3.26E-05 | 7.37E-04 | 0.92 |
| miR-542-3p | 2.12 | 4.06E-05 | 8.80E-04 | 5.52 |
| miR-501-5p | 2.05 | 6.09E-05 | 1.27E-03 | 4.30 |
| miR-146a-5p§ | 1.78 | 7.53E-05 | 1.51E-03 | 5.46 |
| miR-424-5p | 1.94 | 8.69E-05 | 1.63E-03 | 6.68 |
| miR-499a-5p§ | -2.20 | 9.65E-05 | 1.63E-03 | 8.78 |
| miR-4454 | -2.07 | 9.69E-05 | 1.63E-03 | 1.27 |
| miR-3605-3p | -1.75 | 9.70E-05 | 1.63E-03 | 2.14 |
| miR-6724-5p | -2.02 | 9.72E-05 | 1.63E-03 | 2.34 |
| miR-4800-3p | -1.73 | 1.04E-04 | 1.69E-03 | 3.08 |
| miR-208b§ | -1.85 | 1.22E-04 | 1.92E-03 | 10.94 |
| miR-1273g-3p | -1.78 | 1.35E-04 | 2.07E-03 | 1.25 |
| miR-1§ | -1.91 | 1.42E-04 | 2.11E-03 | 11.09 |
| miR-378e | -1.74 | 1.70E-04 | 2.46E-03 | 7.36 |
| miR-30b-3p | -1.84 | 2.20E-04 | 3.09E-03 | 4.39 |
| miR-5699 | -1.85 | 2.27E-04 | 3.10E-03 | 0.17 |
| miR-372 | -1.87 | 2.41E-04 | 3.21E-03 | 0.09 |
| miR-3591-5p | -2.19 | 3.30E-04 | 4.11E-03 | 3.26 |
| miR-329 | 1.96 | 3.32E-04 | 4.11E-03 | 1.45 |
| miR-3960 | -1.77 | 3.32E-04 | 4.11E-03 | 3.66 |
| miR-450a-5p | 1.85 | 3.45E-04 | 4.17E-03 | 5.24 |
| miR-3676-5p | -1.83 | 3.73E-04 | 4.41E-03 | 2.37 |
| miR-503-5p | 1.81 | 3.87E-04 | 4.47E-03 | 2.90 |
| miR-23a-5p | -1.89 | 4.22E-04 | 4.77E-03 | 3.15 |
| miR-133b§ | 1.63 | 4.54E-04 | 5.03E-03 | 12.18 |
| miR-520a-3p | -2.01 | 5.12E-04 | 5.54E-03 | 0.04 |
| miR-196b-5p | 1.60 | 5.72E-04 | 5.99E-03 | 7.71 |
| miR-1271-5p | -1.71 | 5.80E-04 | 5.99E-03 | 5.25 |
| miR-362-3p | 1.67 | 5.88E-04 | 5.99E-03 | 3.38 |
| miR-203a | 1.50 | 6.34E-04 | 6.34E-03 | 3.13 |
| miR-378g | -1.57 | 6.57E-04 | 6.44E-03 | 7.35 |
| miR-450b-5p | 1.76 | 7.06E-04 | 6.71E-03 | 6.18 |
| miR-451a | 1.14 | 7.10E-04 | 6.71E-03 | 12.68 |
| miR-4770 | 1.36 | 7.49E-04 | 6.95E-03 | 3.27 |
| miR-150-5p | -1.70 | 9.58E-04 | 8.74E-03 | 9.08 |
| miR-3913-5p | 1.81 | 9.76E-04 | 8.75E-03 | 0.48 |
| miR-675-5p | -1.29 | 9.99E-04 | 8.80E-03 | 4.41 |
| miR-29b-1-5p | -1.96 | 1.04E-03 | 9.04E-03 | 0.25 |
| miR-143-3p | 1.33 | 1.16E-03 | 9.93E-03 | 16.86 |
| miR-146b-5p | 1.55 | 1.29E-03 | 1.08E-02 | 9.38 |
| miR-378d | -1.54 | 1.35E-03 | 1.12E-02 | 9.34 |
| miR-133a§ | -1.56 | 1.73E-03 | 1.40E-02 | 15.85 |
| miR-193a-5p | -1.50 | 1.99E-03 | 1.60E-02 | 6.81 |
| miR-376c-3p | 1.59 | 2.05E-03 | 1.61E-02 | 5.32 |
| miR-542-5p | 1.63 | 2.17E-03 | 1.68E-02 | 3.51 |
| miR-15b-3p | 1.24 | 2.29E-03 | 1.75E-02 | 2.91 |
| miR-3613-3p | 1.49 | 2.49E-03 | 1.88E-02 | 1.38 |
| miR-335-3p | 1.41 | 2.59E-03 | 1.92E-02 | 6.51 |
| miR-500a-5p | 1.48 | 3.15E-03 | 2.31E-02 | 3.95 |
| miR-324-5p | 1.37 | 3.32E-03 | 2.39E-02 | 4.13 |
| miR-3909 | -1.32 | 3.44E-03 | 2.45E-02 | 3.90 |
| miR-6500-3p | 1.46 | 3.56E-03 | 2.50E-02 | 1.34 |
| miR-616-5p | -1.46 | 4.10E-03 | 2.84E-02 | 2.17 |
| miR-3127-5p | -1.41 | 4.16E-03 | 2.84E-02 | 1.52 |
| miR-486-5p | -1.47 | 4.36E-03 | 2.94E-02 | 17.49 |
| miR-3196 | -1.45 | 4.61E-03 | 3.07E-02 | 1.95 |
| miR-3116 | -1.57 | 5.35E-03 | 3.52E-02 | 1.45 |
| miR-190a | -1.34 | 5.61E-03 | 3.61E-02 | 6.74 |
| miR-1275 | 1.39 | 5.63E-03 | 3.61E-02 | 3.27 |
| miR-4443 | -1.26 | 5.73E-03 | 3.63E-02 | 0.84 |
| miR-24-1-5p | -1.56 | 5.99E-03 | 3.75E-02 | 1.75 |
| miR-378i | -1.42 | 6.31E-03 | 3.91E-02 | 10.15 |
| miR-382-3p | 1.54 | 6.71E-03 | 4.11E-02 | 2.88 |
| miR-6128 | -1.38 | 7.30E-03 | 4.37E-02 | 0.48 |
| miR-21-5p | 1.17 | 7.31E-03 | 4.37E-02 | 12.61 |
| miR-422a | -1.30 | 7.74E-03 | 4.56E-02 | 4.79 |
| miR-143-5p | 1.04 | 7.96E-03 | 4.56E-02 | 4.49 |
| miR-486-3p§ | -1.16 | 7.97E-03 | 4.56E-02 | 7.25 |
| miR-378h | -1.26 | 7.98E-03 | 4.56E-02 | 3.14 |
| miR-181a-5p | 1.08 | 8.34E-03 | 4.71E-02 | 3.52 |
| miR-532-3p | 1.26 | 8.52E-03 | 4.76E-02 | 6.66 |
| miR-136-5p | 1.48 | 8.80E-03 | 4.87E-02 | 7.19 |
| miR-378f | -1.32 | 9.00E-03 | 4.87E-02 | 7.35 |
| miR-28-3p | -1.17 | 9.01E-03 | 4.87E-02 | 11.37 |
| miR-27b-5p | -1.17 | 9.14E-03 | 4.87E-02 | 6.71 |
| miR-660-5p | 1.32 | 9.18E-03 | 4.87E-02 | 9.94 |
| miR-378a-3p | -1.25 | 9.30E-03 | 4.89E-02 | 15.75 |
|  |  |  |  |  |
| **miRNAs differentially expressed in myoblasts** | | | | |
| **hsa-miR** | **LogFC** | **P-value** | **FDR** | **logCPM** |
| miR-204-5p | 6.20 | 1.09E-13 | 6.11E-11 | 7.21 |
| miR-1§ | 5.48 | 1.81E-11 | 5.09E-09 | 4.77 |
| miR-483-5p | 5.30 | 5.57E-11 | 1.05E-08 | 4.42 |
| miR-483-3p | 5.07 | 3.02E-10 | 4.25E-08 | 3.89 |
| miR-133b§ | 4.60 | 2.09E-09 | 2.35E-07 | 6.71 |
| miR-208b§ | 4.24 | 2.15E-08 | 2.01E-06 | 5.17 |
| miR-3607-3p | -3.35 | 6.41E-08 | 5.16E-06 | 2.68 |
| miR-10b-5p | 3.81 | 2.01E-07 | 1.37E-05 | 13.96 |
| miR-206§ | 3.79 | 2.23E-07 | 1.37E-05 | 8.46 |
| miR-4697-3p | -3.32 | 2.43E-07 | 1.37E-05 | 1.04 |
| miR-490-3p | -3.07 | 4.09E-07 | 2.09E-05 | 3.95 |
| miR-184 | 3.49 | 2.92E-06 | 1.37E-04 | 2.60 |
| miR-146a-5p§ | 3.28 | 5.19E-06 | 2.25E-04 | 4.02 |
| miR-133a§ | 3.13 | 9.49E-06 | 3.82E-04 | 8.81 |
| miR-96-5p | -2.52 | 3.00E-05 | 1.13E-03 | 2.23 |
| miR-199b-5p | 2.73 | 7.96E-05 | 2.80E-03 | 6.20 |
| miR-212-3p | -2.15 | 2.75E-04 | 9.11E-03 | 4.32 |
| miR-138-5p | -2.12 | 3.09E-04 | 9.66E-03 | 10.44 |
| miR-618 | -2.04 | 6.78E-04 | 2.01E-02 | 2.35 |
| miR-138-1-3p | -1.94 | 9.83E-04 | 2.77E-02 | 5.37 |
|  |  |  |  |  |
| **miRNAs differentially expressed in myotubes** | | | | |
| **hsa-miR** | **LogFC** | **P-value** | **FDR** | **logCPM** |
| miR-585 | 4.57 | 7.91E-12 | 4.17E-09 | 2.54 |
| miR-208b§ | 3.50 | 1.00E-08 | 2.64E-06 | 7.24 |
| miR-204-5p | 3.23 | 8.31E-08 | 1.46E-05 | 8.33 |
| miR-133b§ | 3.06 | 2.94E-07 | 3.87E-05 | 9.28 |
| miR-1§ | 2.89 | 1.08E-06 | 1.14E-04 | 7.28 |
| miR-133a§ | 2.85 | 1.40E-06 | 1.23E-04 | 11.50 |
| miR-139-5p | 2.77 | 3.11E-06 | 2.34E-04 | 3.39 |
| miR-4697-3p | -2.54 | 9.75E-06 | 6.43E-04 | 0.61 |
| miR-188-3p | 2.40 | 5.99E-05 | 3.51E-03 | 1.67 |
| miR-206§ | 2.16 | 1.63E-04 | 8.60E-03 | 10.51 |
| miR-188-5p | 1.90 | 8.34E-04 | 3.70E-02 | 4.83 |
| miR-501-5p | 1.91 | 8.75E-04 | 3.70E-02 | 3.13 |
| miR-378a-3p | 1.88 | 9.13E-04 | 3.70E-02 | 9.82 |
| miR-3614-5p | -1.80 | 1.33E-03 | 4.99E-02 | 0.51 |
| miR-1268a | 1.80 | 1.58E-03 | 5.00E-02 | 3.46 |
| miR-378f | 1.86 | 1.68E-03 | 5.00E-02 | 1.00 |
| miR-1268b | 1.78 | 1.74E-03 | 5.00E-02 | 3.50 |
| miR-483-5p | 1.77 | 1.77E-03 | 5.00E-02 | 3.89 |
| miR-455-3p | -1.67 | 1.80E-03 | 5.00E-02 | 6.46 |

§: myomiRs
