# Supplementary material for: SMA-miRs (miR-181a-5p, -324-5p, and -451a) are overexpressed in spinal muscular atrophy skeletal muscle and serum samples
Source: eLife. 2021 Sep 20;10:e68054. doi: 10.7554/eLife.68054 (PMC8486378; doi:10.7554/eLife.68054)
Supplement: Supplementary file 5. [file elife-68054-supp5.docx]

**Supplementary Table 5: assessment by relative and/or absolute qPCR of miR levels in serum samples of patients and controls**

| **Assay** | **HSA-miR** | **Relative qPCR**  **SMA vs. CTRL** | | **Absolute qPCR** | | |
| --- | --- | --- | --- | --- | --- | --- |
|  |  | *Fold-change* | *p-value** | *Median levels*  *# of molecules/μl of serum* | | *p-value⸙* |
|  |  |  |  | SMA | CTRL |  |
| r-qPCR | let-7g-3p¹ | Undetectable |  |  |  |  |
| r-qPCR | miR-1271-5p | 2.61 | 0.162 |  |  |  |
| r-qPCR | miR-1275 | Undetectable |  |  |  |  |
| r-qPCR | miR-136-5p | Undetectable |  |  |  |  |
| r-qPCR | miR-143-5p | 2.08 | 0.121 |  |  |  |
| r-qPCR | miR-190a-5p | Undetectable |  |  |  |  |
| r-qPCR | miR-193a-5p | 1.19 | 0.910 |  |  |  |
| r-qPCR | miR-196b-5p | Undetectable |  |  |  |  |
| r-qPCR | miR-203a | 2.94 | 0.076 |  |  |  |
| r-qPCR | miR-204-5p | Undetectable |  |  |  |  |
| r-qPCR | miR-205-5p | 1.65 | 0.345 |  |  |  |
| r-qPCR | miR-208b | 1.89 | 0.121 |  |  |  |
| r-qPCR | miR-21-5p | Undetectable |  |  |  |  |
| r-qPCR | miR-218-5p | 3.07 | 0.064 |  |  |  |
| r-qPCR | miR-224-5p | 4.05 | 0.104 |  |  |  |
| r-qPCR | miR-23a-5p | 1.39 | 0.678 |  |  |  |
| r-qPCR | miR-24-1-5p | Undetectable |  |  |  |  |
| r-qPCR | miR-27b-5p | Undetectable |  |  |  |  |
| r-qPCR | miR-28-3p | Undetectable |  |  |  |  |
| r-qPCR | miR-299-5p | Undetectable |  |  |  |  |
| r-qPCR | miR-30b-3p | Undetectable |  |  |  |  |
| r-qPCR | miR-3116 | Undetectable |  |  |  |  |
| r-qPCR | miR-3127-5p | Undetectable |  |  |  |  |
| r-qPCR | miR-335-3p | 2.23 | 0.064 |  |  |  |
| r-qPCR | miR-338-5p | 1.22 | 0.910 |  |  |  |
| r-qPCR | miR-378f | Undetectable |  |  |  |  |
| r-qPCR | miR-378h | Undetectable |  |  |  |  |
| r-qPCR | miR-378i | Undetectable |  |  |  |  |
| r-qPCR | miR-382-3p | Undetectable |  |  |  |  |
| r-qPCR | miR-422a | Undetectable |  |  |  |  |
| r-qPCR | miR-424-5p | 1.20 | 0.910 |  |  |  |
| r-qPCR | miR-450a-5p | Undetectable |  |  |  |  |
| r-qPCR | miR-450b-5p | 0.60 | 0.678 |  |  |  |
| r-qPCR | miR-452-5p | 1.03 | 0.910 |  |  |  |
| r-qPCR | miR-486-5p | Undetectable |  |  |  |  |
| r-qPCR | miR-499a-5p | 1.19 | 0.734 |  |  |  |
| r-qPCR | miR-500a-5p | Undetectable |  |  |  |  |
| r-qPCR | miR-501-5p | 2.94 | 1.000 |  |  |  |
| r-qPCR | miR-503-5p | Undetectable |  |  |  |  |
| r-qPCR | miR-512-3p | 0.97 | 0.678 |  |  |  |
| r-qPCR | miR-516b-5p¹ | 0.89 | 0.571 |  |  |  |
| r-qPCR | miR-520a-3p | Undetectable |  |  |  |  |
| r-qPCR | miR-532-3p | Undetectable |  |  |  |  |
| r-qPCR | miR-542-5p | 6.63 | 0.065 |  |  |  |
| r-qPCR | miR-5699-3p | Undetectable |  |  |  |  |
| r-qPCR | miR-6128 | Undetectable |  |  |  |  |
| r-qPCR | miR-616-5p | Undetectable |  |  |  |  |
| r-qPCR | miR-660-5p | Undetectable |  |  |  |  |
| r-qPCR | miR-885-5p | 0.19 | 1.000 |  |  |  |
| r-qPCR | miR-95 | 3.26 | 0.089 |  |  |  |
| r-qPCR & a-qPCR | miR-1 | Undetectable |  | Undetectable | Undetectable |  |
| r-qPCR & a-qPCR | miR-133a | 34.44 | *<0.000* | 96.32 | 75.18 | 0.100 |
| r-qPCR & a-qPCR | miR-133b | 16.31 | *0.001* | 23.78 | 24.46 | 0.420 |
| r-qPCR & a-qPCR | miR-143-3p | 13.28 | *0.001* | 30.08 | 33.29 | 0.330 |
| r-qPCR & a-qPCR | miR-145-5p | 3.92 | *0.002* | 11.21 | 9.58 | 0.400 |
| r-qPCR & a-qPCR | miR-146a-5p | 2.52 | *0.014* | 0.21 | 2.35 | 0.100 |
| r-qPCR & a-qPCR | miR-146b-5p | 5.17 | *0.026* | 29.92 | 24.44 | 0.390 |
| r-qPCR & a-qPCR | miR-15b-3p | 1.41 | *0.064* | 115.85 | 95.80 | 0.120 |
| r-qPCR & a-qPCR | **miR-181a-5p** | **8.16** | ***<0.000*** | **136.20** | **51.56** | ***<0.000*** |
| r-qPCR & a-qPCR | miR-19a-3p | 6.84 | *0.004* | 7.06 | 5.90 | 0.420 |
| r-qPCR & a-qPCR | miR-19b-3p | 3.67 | *0.004* | 8.60 | 8.22 | 0.490 |
| r-qPCR & a-qPCR | miR-206¹ | 8.11 | *0.003* | 69.81 | 88.56 | 0.250 |
| r-qPCR & a-qPCR | miR-215 | 3.94 | *0.001* | 3.68 | 8.52 | 0.330 |
| r-qPCR & a-qPCR | miR-23a-3p¹ | 13.17 | *0.006* | 194.14 | 275.65 | 0.730 |
| r-qPCR & a-qPCR | miR-26a-5p¹ | 21.21 | *0.002* | Undetectable | Undetectable |  |
| r-qPCR & a-qPCR | miR-29b-1-5p | 0.00 | *0.002* | Undetectable | Undetectable |  |
| r-qPCR & a-qPCR | **miR-324-5p** | **8.40** | ***<0.000*** | **206.20** | **152.30** | ***0.020*** |
| r-qPCR & a-qPCR | miR-329 | 12.39 | *<0.000* | Undetectable | Undetectable |  |
| r-qPCR & a-qPCR | miR-335-5p | 2.05 | *0.016* | 245.17 | 202.47 | 0.410 |
| r-qPCR & a-qPCR | miR-362-3p | 3.14 | *0.014* | 30.17 | 14.03 | 0.160 |
| r-qPCR & a-qPCR | miR-376c-3p | 12.31 | *0.002* | 16.82 | 11.26 | 0.130 |
| r-qPCR & a-qPCR | **miR-451a** | **6.63** | ***0.002*** | **69.69** | **26.73** | ***0.004*** |
| r-qPCR & a-qPCR | miR-499a-3p | 0.00 | *0.001* | 161.53 | 99.71 | 0.170 |
| r-qPCR & a-qPCR | let-7c¹ | 9.69 | *<0.000* | 6.96 | 11.50 | 0.100 |
| a-qPCR | miR-1273g-3p |  |  | Undetectable | Undetectable |  |
| a-qPCR | miR-1303 |  |  | Undetectable | Undetectable |  |
| a-qPCR | miR-1469 |  |  | Undetectable | Undetectable |  |
| a-qPCR | miR-150-5p |  |  | 153.66 | 49.82 | 0.110 |
| a-qPCR | miR-3196 |  |  | Undetectable | Undetectable |  |
| a-qPCR | miR-3591-5p |  |  | Undetectable | Undetectable |  |
| a-qPCR | miR-3605-3p |  |  | Undetectable | Undetectable |  |
| a-qPCR | miR-3613-3p |  |  | Undetectable | Undetectable |  |
| a-qPCR | miR-378d |  |  | Undetectable | Undetectable |  |
| a-qPCR | miR-378e |  |  | Undetectable | Undetectable |  |
| a-qPCR | miR-3909 |  |  | 3.62 | 25.21 |  |
| a-qPCR | miR-3913-5p |  |  | Undetectable | Undetectable |  |
| a-qPCR | miR-4443 |  |  | Undetectable | Undetectable |  |
| a-qPCR | miR-4454 |  |  | Undetectable | Undetectable |  |
| a-qPCR | miR-4800-3p |  |  | Undetectable | Undetectable |  |
| a-qPCR | miR-5690 |  |  | Undetectable | Undetectable |  |
| a-qPCR | miR-6724-5p |  |  | Undetectable | Undetectable |  |
| a-qPCR | miR-1281 |  |  | 4.82 | 3.89 | 0.350 |
| a-qPCR | miR-184 |  |  | Undetectable | Undetectable |  |
| a-qPCR | miR-3676-5p |  |  | Undetectable | Undetectable |  |
| a-qPCR | miR-372 |  |  | Undetectable | Undetectable |  |
| a-qPCR | miR-378a-3p |  |  | Undetectable | Undetectable |  |
| a-qPCR | miR-378g |  |  | 36.29 | 23.57 | 0.090 |
| a-qPCR | miR-3960 |  |  | 5.73 | 15.78 | 0.100 |
| a-qPCR | miR-4532 |  |  | 18.20 | 8.23 | 0.080 |
| a-qPCR | miR-4770 |  |  | 30.78 | 37.64 | 0.290 |
| a-qPCR | miR-486-3p |  |  | Undetectable | Undetectable |  |
| a-qPCR | miR-542-3p |  |  | Undetectable | Undetectable |  |
| a-qPCR | miR-6500-3p |  |  | 2.71 | 3.19 | 0.380 |
| a-qPCR | miR-655 |  |  | Undetectable | Undetectable |  |
| a-qPCR | miR-675-5p |  |  | Undetectable | Undetectable |  |
| a-qPCR | miR-30c-5p¹ |  |  | 3012.89 | 437.44 | 0.310 |
| a-qPCR | miR-339-5p¹ |  |  | 475.26 | 486.71 | 0.600 |
| a-qPCR | miR-9-3p¹ |  |  | 8.22 | 8.52 | 0.300 |
| a-qPCR | miR 16a-5p¹ |  |  | 8.13 | 4.96 | 0.060 |
| a-qPCR | miR-181a2-3p¹ |  |  | 6.48 | 9.00 | 0.320 |

*: p-values refer to the significance of comparison of the miR levels in patients vs. controls by Benjamini-Hochberg False Discovery Rate (FDR) method. P-values<0.05 were considered significant

*⸙*: p-values refer to the significance of comparison of the miR levels in patients vs. controls by Mann-Withney U-test. P-values<0.05 were considered significant.
