# Supplementary material for: SMA-miRs (miR-181a-5p, -324-5p, and -451a) are overexpressed in spinal muscular atrophy skeletal muscle and serum samples
Source: eLife. 2021 Sep 20;10:e68054. doi: 10.7554/eLife.68054 (PMC8486378; doi:10.7554/eLife.68054)
Supplement: Transparent reporting form [file elife-68054-transrepform1.docx]

***eLife’s* transparent reporting form**

We encourage authors to provide detailed information *within their submission* to facilitate the interpretation and replication of experiments. Authors can upload supporting documentation to indicate the use of appropriate reporting guidelines for health-related research (see [EQUATOR Network](http://www.equator-network.org/%20)), life science research (see the [BioSharing Information Resource](https://biosharing.org/)), or the [ARRIVE guidelines](http://www.plosbiology.org/article/info:doi/10.1371/journal.pbio.1000412) for reporting work involving animal research. Where applicable, authors should refer to any relevant reporting standards documents in this form.

If you have any questions, please consult our Journal Policies and/or contact us: [editorial@elifesciences.org](mailto:editorial@elifesciences.org).

**Sample-size estimation**

- You should state whether an appropriate sample size was computed when the study was being designed
- You should state the statistical method of sample size computation and any required assumptions
- If no explicit power analysis was used, you should describe how you decided what sample (replicate) size (number) to use

Please outline where this information can be found within the submission (e.g., sections or figure legends), or explain why this information doesn’t apply to your submission:

At the time of study design, no preliminary data on the outcome of the whole miRNome analysis were available. For this reason, a formal sample size evaluation was not feasible. Since muscle biopsies of SMA patients are very precious and rare samples, to maximize the outcome of the study we opted for testing all samples available in the biobank that fulfilled the selection criteria, as specified in the manuscript. A similar reasoning was followed for muscle cell cultures, that are even more difficultly available. However, since our study led to highly significant results, the number of samples analysed was adequate for our purposes.

Regarding serum samples, similar studies that were published over the last few years have been performed in markedly smaller cohorts. Also in this case, at the time of study design, preliminary data were not available.

**Replicates**

- You should report how often each experiment was performed
- You should include a definition of biological versus technical replication
- The data obtained should be provided and sufficient information should be provided to indicate the number of independent biological and/or technical replicates
- If you encountered any outliers, you should describe how these were handled
- Criteria for exclusion/inclusion of data should be clearly stated
- High-throughput sequence data should be uploaded before submission, with a private link for reviewers provided (these are available from both GEO and ArrayExpress)

Please outline where this information can be found within the submission (e.g., sections or figure legends), or explain why this information doesn’t apply to your submission:

The details above are specified in the text. For whole miRNome analysis, the different samples were considered as biological replicates and used for grouping (patients vs. controls). Due to the cost of the assay, no technical replicates were performed: however, in this approach, to our knowledge, technical replicates in –omics approaches are not the rule.

For serum samples analysis, each sample was analysed at least twice in triplicates for each experiment. For shortness, this aspect (that is usual in our laboratory settings, as stated in our previous works) was not specified in the manuscript, but if required the text can be modified accordingly.

Regarding possible outliers, all samples were included in the analysis, as it appears from the boxplots of the manuscripts.

Regarding, high throughput sequencing data, could you please specify if you are referring to the raw sequencing data (.bam and .bai files) or to the outcome of the analysis? This second dataset is already included in the manuscript as supplementary table. We are in the process of recovering raw sequencing data from our bioinformatics. This will require a bit longer. However, these data are likely to be more useful to the scientific community for possible future re-analyses or meta-analyses, rather than for the reviewing process.

**Statistical reporting**

- Statistical analysis methods should be described and justified
- Raw data should be presented in figures whenever informative to do so (typically when N per group is less than 10)
- For each experiment, you should identify the statistical tests used, exact values of N, definitions of center, methods of multiple test correction, and dispersion and precision measures (e.g., mean, median, SD, SEM, confidence intervals; and, for the major substantive results, a measure of effect size (e.g., Pearson's r, Cohen's d)
- Report exact p-values wherever possible alongside the summary statistics and 95% confidence intervals. These should be reported for all key questions and not only when the p-value is less than 0.05.

Please outline where this information can be found within the submission (e.g., sections or figure legends), or explain why this information doesn’t apply to your submission:

All these aspects have been reported in the manuscript, either in Methods section or in the Results/figure legends

(For large datasets, or papers with a very large number of statistical tests, you may upload a single table file with tests, Ns, etc., with reference to sections in the manuscript.)

**Group allocation**

- Indicate how samples were allocated into experimental groups (in the case of clinical studies, please specify allocation to treatment method); if randomization was used, please also state if restricted randomization was applied
- Indicate if masking was used during group allocation, data collection and/or data analysis

Please outline where this information can be found within the submission (e.g., sections or figure legends), or explain why this information doesn’t apply to your submission:

This has been clearly stated in methods. Dealing with patients affected from a genetic disorder, the two classes of patients and controls are dichotomous, with no risk of overlapping.

**Additional data files (“source data”)**

- We encourage you to upload relevant additional data files, such as numerical data that are represented as a graph in a figure, or as a summary table
- Where provided, these should be in the most useful format, and they can be uploaded as “Source data” files linked to a main figure or table
- Include model definition files including the full list of parameters used
- Include code used for data analysis (e.g., R, MatLab)
- Avoid stating that data files are “available upon request”

Please indicate the figures or tables for which source data files have been provided:

The excel file containing individual results will be uploaded.

In the manuscript, the statement “data not shown” or “data available on request” is present in the Results section and refers to ancillary or negative data that are not of relevance with respect to the study as a whole. For this reason we preferred not to include either in supplemental materials. If the editorial board and/or any of the reviewers would feel that these date may provide any additional value to the study, we are completely available for sharing.
